# Prognostic Value of the Six-Second Spirometry in Patients with Chronic Obstructive Pulmonary Disease: A Cohort Study

**DOI:** 10.1371/journal.pone.0140855

**Published:** 2015-10-21

**Authors:** Eva Prats, Elena Tejero, Paloma Pardo, Adelaida Gavilán, Raúl Galera, José Ramón Donado, Miguel Ángel Racionero, Raquel Casitas, Antonio Zapatero, Francisco García-Río

**Affiliations:** 1 Unidad de Neumología. Hospital Universitario de Fuenlabrada, Fuenlabrada, Madrid, Spain; 2 Servicio de Urgencias. Hospital Universitario de Fuenlabrada, Fuenlabrada, Madrid, Spain; 3 Servicio de Neumología. Hospital Universitario La Paz, IdiPAZ, Madrid, Spain; 4 Servicio de Medicina Interna. Hospital Universitario de Fuenlabrada, Fuenlabrada, Madrid, Spain; 5 CIBER de Enfermedades Respiratorias (CIBERES), Madrid, Spain; 6 Universidad Autónoma de Madrid, Madrid, Spain; University of Athens, GREECE

## Abstract

**Background:**

The six-second spirometry has been proposed as an alternative to diagnose airflow limitation, although its prognostic value in patients with chronic obstructive pulmonary disease (COPD) remains unknown. The purpose of this study was to determine the prognostic value of the postbronchodilator forced expiratory volume in 1 second (FEV_1_)/forced expiratory volume in 6 seconds (FEV_6_) ratio and FEV_6_ in COPD patients.

**Methods and Findings:**

The study population consisted of 2,614 consecutive stable patients with COPD. The patients were monitored for an average period of 4.3 years regarding mortality, hospitalizations by COPD exacerbations, diagnosis of lung cancer, and annual lung function decline. The overall rate of death was 10.7 (95%CI: 8.7–12.7) per 1000 person-years. In addition to male gender, age and comorbidity, FEV_6_ (hazard ratio [HR]: 0.981, 95%CI: 0.968–0.003) and FEV_1_/FEV_6_ quartiles (lowest quartile (<74% pred.): HR 3.558, 95%CI: 1.752–7.224; and second quartile (74–84% pred.): HR 2.599, 95%CI: 1.215–5.561; versus best quartile (>0.89% pred.)) were independently associated with mortality, whereas FEV_1_ was not retained in the model. 809 patients (30.9%) had at least one hospital admission due to COPD exacerbation. In addition to sex, age, smoking and comorbidity, FEV_1_ and FEV_1_/FEV_6_ quartiles were independent risk factors of hospitalization. FEV_6_ was the only spirometric parameter independently related with lung function annual decline, while the FEV_6_ and FEV_1_/FEV_6_ quartiles were independent risk factors for lung cancer.

**Conclusions:**

In a general COPD outpatient population, airflow obstruction assessed by the FEV_1_/FEV_6_ is an independent risk factor for both death and hospitalization.

## Introduction

Chronic obstructive pulmonary disease (COPD) is the third leading cause of death worldwide and the ninth combining the years of life lost or lived with disability [1]. Since its prevalence and mortality are still increasing, it constitutes a relevant public health problem [2]. COPD is characterized by airflow limitation and therefore spirometry remains the essential test to diagnose and assess the severity of the disease. Although several multidimensional indices have shown better survival prediction than the degree of airflow limitation degree [3,4], all indices have been constructed by adding different variables–such as dyspnoea, exercise capacity, exacerbations or age–to different categories of airflow limitation. Indeed, the new GOLD stratification of COPD severity also includes the level of daily symptoms and the history of exacerbations, along with degree of airflow limitation [2].

Although the forced expiratory volume at 1 second (FEV_1_)/forced vital capacity (FVC) ratio is the gold standard to identify airway limitation, its severity is usually assessed by FEV_1_[2]. In fact, spirometry can require prolonged expiratory effort (which can surpass 20 seconds) to achieve a plateau on the volume-time curve and a small end-of-test volume, which indicates complete lung emptying [5]. With slow lung emptying, as especially occurs in patients with airflow limitation, FVC is sensitive to expiratory time: the longer the expiratory time, the higher the FVC and the smaller the FEV_1_/FVC [6].

Forced expiratory volume in 6 seconds (FEV_6_) has been proposed as a simplified alternative to FVC [7]. FEV_6_ measurement is more easily achieved, causes less patient discomfort and is more reproducible than FVC [8]. Indeed, the FEV_1_/FEV_6_ ratio has been found to be nearly equivalent to FEV_1_/FVC for the diagnosis of airflow limitation [8,9]. Moreover, a meta-analysis indicated that FEV_1_/FEV_6_ can be used as a surrogate for FEV_1_/FVC to quantitate airflow limitation [10].

Having accepted its diagnostic utility, it seems interesting to evaluate whether the FEV_1_/FEV_6_ ratio might have an additional prognostic value in COPD patients as a marker of the degree of airflow limitation. Some previous evidence in smokers without airflow limitation suggests that the FEV_1_/FEV_6_ ratio might be an independent predictor for annual decline in lung function [11]. Moreover, in a cohort of elderly subjects with or without airflow limitation, Sorino et al [12] reported that the FEV_1_/FEV_6_ ratio should be an independent predictor of mortality, with a value comparable to that of FEV_1_ but with higher repeatability. More recently, in a population-based study, it has been reported that the presence of COPD defined by an FEV_1_/FEV_6_ ratio < lower limit of normal was associated with higher overall mortality [13]. Finally, as airflow limitation has been related to higher lung cancer risk [14,15], the FEV_1_/FEV_6_ ratio, as a surrogate measurement of airflow limitation, might also be a risk factor for developing lung cancer.

Therefore, the aim of the present study was to evaluate the prognostic value of the postbronchodilator FEV_6_ and the FEV_1_/FEV_6_ ratio as percentage of predicted (in quartiles) as alternative indicators of airflow limitation in COPD patients.

## Methods

### Study design

We conducted a single-centre, observational cohort study at the Fuenlabrada Hospital, Spain. This is the only community hospital for the 9th district of the Madrid Metropolitan Area, with a population of approximately 215,000 inhabitants. The ethics committee of Area 9 (Hospital Severo Ochoa-Hospital de Fuenlabrada) has approved the study protocol and procedures. Written informed consent was not given by participants for their clinical records to be used in this study, but patient records/information was anonymized and de-identified prior to analysis.

### Study population

The study population consists of COPD patients who were being treated by a general practitioner or pulmonologist. All consecutive subjects aged 40 year or older who had been sent for spirometry between April 1, 2004 and December 31, 2008 were screened, and we recruited those who met the following inclusion criteria: 1) stable clinical condition, with no respiratory infection in the previous 6 weeks, 2) postbronchodilator FEV_1_/ FVC ratio <0·7 and <lower limit of normal, and 3) diagnosis of COPD in the patient’s clinical record, corresponding with the chronic bronchitis (491·xx) or emphysema (492·xx) codes of the International Classification of Diseases, Ninth Revision, Clinical Modification (ICD-9-CM). To minimize the potential misclassification of acute bronchitis as COPD, we did not include unspecified bronchitis (490·xx).

**Fig 1:**
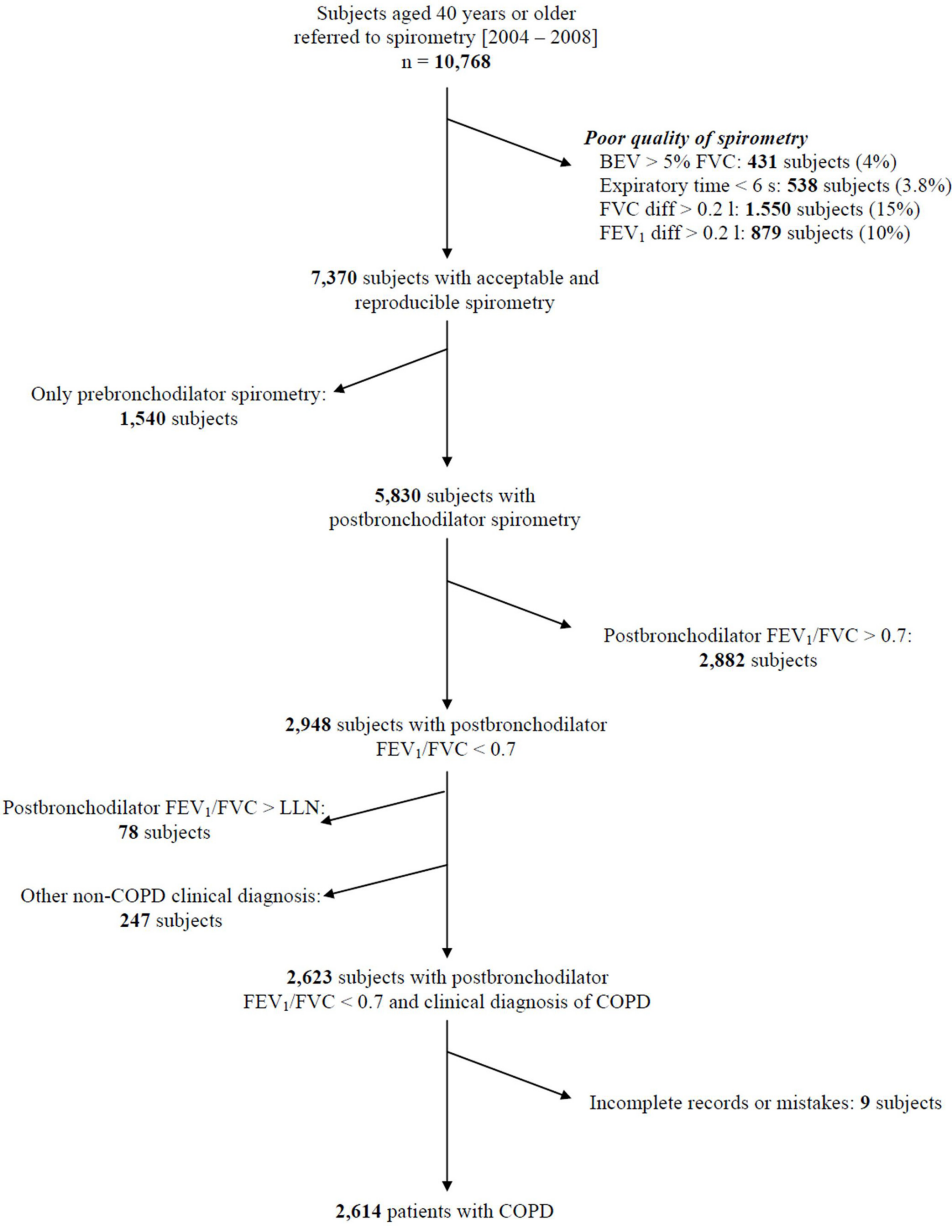
Flow chart of the study recruitment.

Exclusion criteria were: inability to obtain acceptable and reproducible spirometric measurements according to ATS/ERS recommendations [5]; diagnosis of asthma, cystic fibrosis, interstitial lung disease, pulmonary thromboembolic disease, active tuberculosis, chest wall disease, neuromuscular disorder, or malignant tumour; or history of thoracotomy with pulmonary resection, uncontrolled or serious diseases, or other symptoms that could potentially affect the spirometry test. Participants who received antibiotics and/or steroids in the month prior to the enrolment were also excluded. A flowchart of the recruitment process is presented in Fig 1.

### Sample size calculation

Sample size was estimated to compare the hospitalization rates among the postbronchodilator FEV_1_/FEV_6_ quartiles. In patients with mild-to-severe COPD, a hospitalization/year rate of 0·28 ± 0·034 has been previously described in our country [16]. Thus, to detect an inter-group difference of 0·06 hospitalizations/year using two-sided analysis with an alpha error of 0·05, a beta error of 0·20 and 22% of drop-outs, 348 patients were necessary in each subgroup. According to the distribution of FEV_1_/FEV_6_ ratio in our area, a total of at least 2,592 patients were necessary.

### Procedures

Anthropometric characteristics, smoking habit and baseline therapy (inhaled short-acting or long-acting beta-agonist, short-acting or long-acting anticholinergic, oral or inhaled corticosteroid, theophylline, N-acetyl cysteine, and/or long-term home oxygen therapy) were recorded for all patients. Spirometries were performed by the same technician with a MasterScreen Body (Jaeger-Viasys, Würtzburg, Germany), following current guidelines [5]. FVC, FEV_1_, and FEV_6_ were automatically selected as the best value of three acceptable, reproducible manoeuvres [5]. After baseline evaluation, four separate doses of 100 mg of salbutamol were given by metered dose inhaler using a spacer and spirometry was repeated after 15-min delay. Both for the baseline examination as well as the follow-up visits, we only accepted the spirometries with quality grades A or B (three ATS/ERS acceptable maneuvers and a difference less than or equal to 0.2 l between the 2 best FVC and FEV_1_).

As reference values, NHANES predictive equations were used [17]. Additional variables were collected from medical records, including baseline severity of airflow limitation according to the GOLD classification for FEV_1_%pred. [18]; we likewise recorded presence of diabetes, hypertension, ischemic heart disease and/or valve disease, cor pulmonale, hepatic disease, peptic ulcer disease, psychiatric disorders, rheumatic disease, any history of stroke or deep-vein thrombosis, and any other conditions needed to determine the Charlson comorbidity index.

### Follow-up and outcome measurements

Patients were treated by their general practitioner or pulmonologist according to current guidelines [18], and they were checked every 3–6 months during the follow-up period until December 31, 2009. We recorded the changes in smoking habit, comorbidity, and current treatment. The interval between spirometries during the follow-up period was established by clinical indication.

The main outcomes measured were all-cause mortality and hospitalization due to COPD exacerbation. Exacerbation of COPD was defined as an increase in at least 2 out of 3 specified symptoms (breathlessness, sputum volume, sputum purulence) requiring an urgent visit to the emergency department for additional treatment, with ICD-9-CM codes 491·21 or 491·22. Other outcome measurements of interest were hospital admission due to pneumonia (ICD-9-CM codes 480–486), diagnosis of lung cancer (M alphanumeric codes of ICD-9-CM), and annual lung function decline. Vital status and hospitalizations were ascertained by follow-up visits, emergency department or general practitioner reports, phone contacts and clinical records. A participant was considered lost to follow-up if we could not contact the patient or if he or she had moved to another place. Results were reported for patients within a minimum follow-up of 12 months in cases of lung function decline, or within 3 months for the other cases.

### Statistical analysis

Values are expressed as mean ± SD or percentage. Differences between study groups were analysed using ANOVA with Bonferroni post-hoc analysis, Student t or chi-square tests. Relationships between variables were evaluated by Pearson correlation and multiple linear regression or multiple logistic regression models.

Kaplan-Meier curves and log rank tests of both mortality and hospitalizations were performed after stratifying by analysis subgroups. On multivariate Cox regression analysis, variables were included if they were independently associated with both the outcome and the exposure (p < 0·05) or if they modified the risk ratio estimate for any of the remaining covariates (> 0·5% change). Survival models were always adjusted for age, sex, pack-years, body mass index, Charlson index and current treatment. As an additional analysis, Poisson regression with overdispersion correction by Pearson were used to assess the significance of the weighted rate ratios for hospitalization.

All effects were considered significant with a p value < 0·05. Statistical analyses were performed using the Statistical Package for the Social Sciences, v13.0 (SPSS Inc, Chicago, IL, USA) and SAS for Windows statistical software, v9.2 (SAS Institute, Inc., Carey, NC, USA).

## Results

The general characteristics of the 2,614 stable COPD patients included in the study are given in Table 1. The quartile distribution of the postbronchodilator FEV_1_/FEV_6_ ratio (% pred.) was < 74% pred. (quartile 1), 74–84% pred. (quartile 2), 84–89% pred. (quartile 3), and > 89% pred. (quartile 4).

**Table 1:** General characteristics of the study subjects*.

		Mild COPD patients	Moderate COPD patients	Severe COPD patients	Very severe COPD patients	Total COPD patients
N		552	1448	523	91	2614
Males, %		73.2	69.4	71.5	77.8	70.9
Age, yrs		63 ± 13	63 ± 12	66 ± 11	64 ± 10	64 ± 12
Height, m		1.63 ± 0.09	1.63 ± 0.09	1.61 ± 0.09	1.63 ± 0.08	1.62 ± 0.09
BMI, Kg/m^2^		28.0 ± 5.0	29.3 ± 5.6	28.6 ± 6.2	26.1 ± 5.6	28.8 ± 5.6
Smoking status						
	Current smokers, %	37.5	36.0	33.1	32.5	35.6
	Ex–smokers, %	41.0	43.5	49.4	53.8	44.5
	Never smokers, %	21.5	20.4	17.5	13.8	19.8
Pack-years		39.9 ± 24.2	48.1 ± 27.4	54.5 ± 27.8	51.3 ± 24.0	47.9 ± 27.2
Comorbidity						
	Ischemic heart disease, %	6.2	7.1	6.0	6.7	6.6
	Congestive heart failure, %	3.4	6.1	12.1	5.6	6.7
	Cerebrovascular disease, %	4.2	3.6	2.9	2.2	3.5
	Diabetes, %	10.5	15.1	18.6	9.0	14.6
Charlson index		3.8 ± 2.2	3.9 ± 2.1	4.3 ± 2.1	3.8 ± 1.7	3.9 ± 2.1
Lung function						
	Postbronchodilator FVC, % pred.	101 ± 11	79 ± 12	60 ± 11	47 ± 12	78 ± 19
	Postbronchodilator FEV_6_, % pred.	102 ± 11	79 ± 10	60 ± 9	46 ± 8	79 ± 18
	Postbronchodilator FEV_1_, % pred.	90 ± 9	65 ± 8	41 ± 6	26 ± 4	64 ± 19
	Postbronchodilator FEV_1_/FVC	0.67 ± 0.04	0.63 ± 0.07	0.53 ± 0.10	0.43 ± 0.12	0.61 ± 0.09
	Postbronchodilator FEV_1_/FVC, % pred.	88 ± 5	84 ± 8	73 ± 13	60 ± 15	81 ± 12
	Postbronchodilator FEV_1_/FEV_6_	0.70 ± 0.04	0.65 ± 0.07	0.56 ± 0.09	0.47 ± 0.08	0.64 ± 0.09
	Postbronchodilator FEV_1_/FEV_6_, % pred.	88 ± 5	84 ± 7	73 ± 11	60 ± 10	81 ± 11
Current treatment						
	SABA, %	36.5	43.9	50.1	56.2	44.0
	LABA, %	37.6	63.4	80.0	80.9	61.9
	SAMA, %	4.4	7.3	14.2	9.0	8.1
	LAMA, %	24.3	50.1	65.5	75.3	48.6
	Theophyllines, %	0.9	2.5	10.2	25.8	4.5
	Inhaled corticosteroids, %	39.2	61.9	79.7	82.0	61.3
	NAC, %	4.5	5.4	8.8	4.5	5.9
	LTOT, %	2.7	7.1	23.4	37.1	10.5

*Data are mean ± SD or percentage.

Definition of abbreviations: BMI = body mass index; FVC = forced vital capacity; FEV_6_ = forced expiratory volume in 6 seconds; FEV_1_ = forced expiratory volume in 1 second; SABA = short-acting betaadrenergic agonists; LABA = long-acting betaadrenergic agonists; SAMA = short acting muscarinic antagonist; LAMA = long acting muscarinic antagonist; NAC = N-acetylcysteine; LTOT = long-term oxygen therapy.

### Prediction of mortality

**Fig 2:**
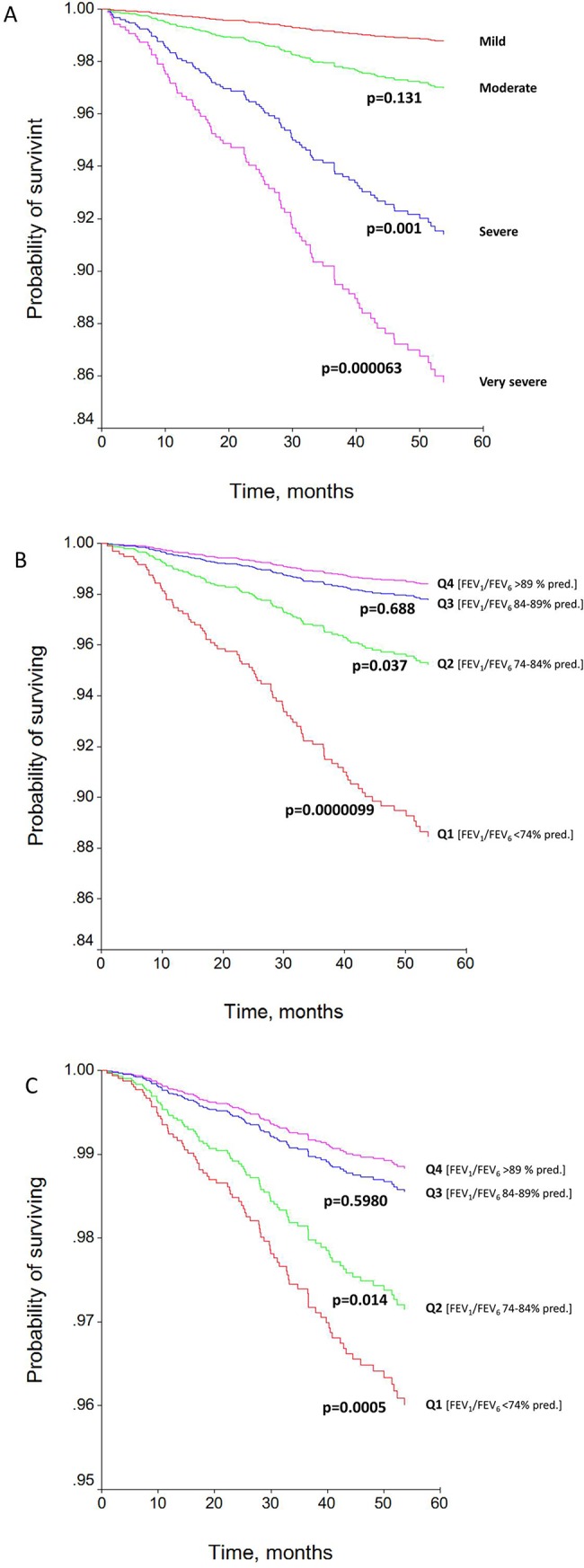
Crude mortality risk of COPD patients classified according to degree of airflow limitation (A) and the quartiles of postbronchodilator FEV_1_/FEV_6_ ratio (B). Adjusted hazard ratio for quartiles of postbronchodilator FEV_1_/FEV_6_, derived from the stepwise regression model, is shown in C.

Ninety-seven of the 2,614 evaluated patients (3·7%) died during the follow-up period of 51 ± 14 months. This represents an overall death rate of 10·7 (95CI: 8·7–12·7) per 1000 person-years. S1 Table compares the characteristics of survivors and nonsurvivors. The patients who died were predominantly males, older and heavier smokers who had lower body mass index, higher comorbidity and greater lung function impairment than survivor patients. Fig 2 shows the survival curves according to the degree of airflow limitation and the quartiles of postbronchodilator FEV_1_/FEV_6_%pred. Time to death was shorter in patients with COPD and lower levels of both parameters.

Table 2 shows the influence on prognosis of the variables included in the univariate survival analysis. After adjusting for all relevant confounders, significant hazard ratios (HRs) were observed for the degree of airflow limitation as well as postbronchodilator FVC%pred., FEV_6_%pred., and FEV_1_/FEV_6_ ratio (%pred.). Finally, in the stepwise multivariate Cox regression model, only male sex, age, comorbidity, postbronchodilator FEV_6_%pred., and quartiles of postbronchodilator FEV_1_/FEV_6_%pred. were retained as independent predictors of mortality (Table 2, Fig 2C). In contrast, airflow limitation severity, assessed by postbronchodilator FEV_1_%pred., was not retained in the model.

**Table 2:** Risk factors for mortality in COPD patients.

		*Crude hazard ratio (95% CI)*	*p*	*Model 1* *		*Model 2†*	
				*Adjusted hazard ratio (95% CI)*	*p*	*Adjusted hazard ratio (95%CI)*	*p*
Males (vs. females)		13.898 (4.084–40.729)	<0.001	-	-	9.056 (2.839–28.894)	0.0002
Age, yrs.		1.067 (1.047–1.088)	<0.001	-	-	1.060 (1.029–0.092)	0.0001
BMI, Kg/m^2^		0.932 (0.896–0.969)	<0.001	-	-		
Pack-years		1.015 (1.008–1.022)	<0.001	-	-		
Charlson morbidity index		1.272 (1.187–1.362)	<0.001	-	-	1.162 (1.020–1.325)	0.024
Airflow limitation severity (GOLD)			<0.001		<0.001		
	Mild (n = 343)	1	-	1	-		
	Moderate (n = 1433)	2.491 (0.762–8.148)	0.131	1.659 (0.501–5.490)	0.407		
	Severe (n = 690)	7.267 (2.258–23.386)	0.001	3.615 (1.105–11.819)	0.034		
	Very severe (n = 148)	12.403 (3.614–42.568)	<0.001	5.247 (1.506–18.276)	0.009		
Postbronchodilator FVC, % pred.		0.968 (0.958–0.979)	<0.001	0.981 (0.969–0.992)	0.001		
Postbronchodilator FEV_6_, % pred.		0.553 (0.425–0.719)	<0.001	0.973 (0.961–0.985)	<0.001	0.981 (0.968–0.993)	0.003
Postbronchodilator FEV_1_/FEV_6_, % pred.			<0.001		<0.001	-	0.001
	Q4 (>0.89% pred.) (n = 571)	1	-	1	-	1	-
	Q3 (82–89% pred.) (n = 570)	0.830 (0.334–2.063)	0.688	1.263 (0.497–3.213)	0.624	1.285 (0.506–3.264)	0.598
	Q2 (74–84% pred.) (n = 570)	2.164 (1.050–4.464)	0.037	2.711 (1.265–5.808)	0.010	2.599 (1.215–5.561)	0.014
	Q1 (< 74% pred.) (n = 570)	4.408 (2.283–8.511)	<0.001	3.897 (1.911–7.946)	<0.001	3.558 (1.752–7.224)	0.0005

97 patients died during the follow-up period (3.7%)

*Adjusted for age, sex, body mass index, smoking status, Charlson morbidity index and current treatment.

†Stepwise multivariate model including age, sex, body mass index, smoking status, Charlson morbidity index, current treatment, airflow limitation severity and postbronchodilator values of VC, FEV_6_ and FEV_1_/FEV_6_.

Abbreviatures: FVC = forced vital capacity; FEV_1_ = forced expiratory volume in 1 second; FEV_6_ = forced expiratory volume in 6 seconds.

### Prediction of hospitalization due to COPD exacerbations

**Fig 3:**
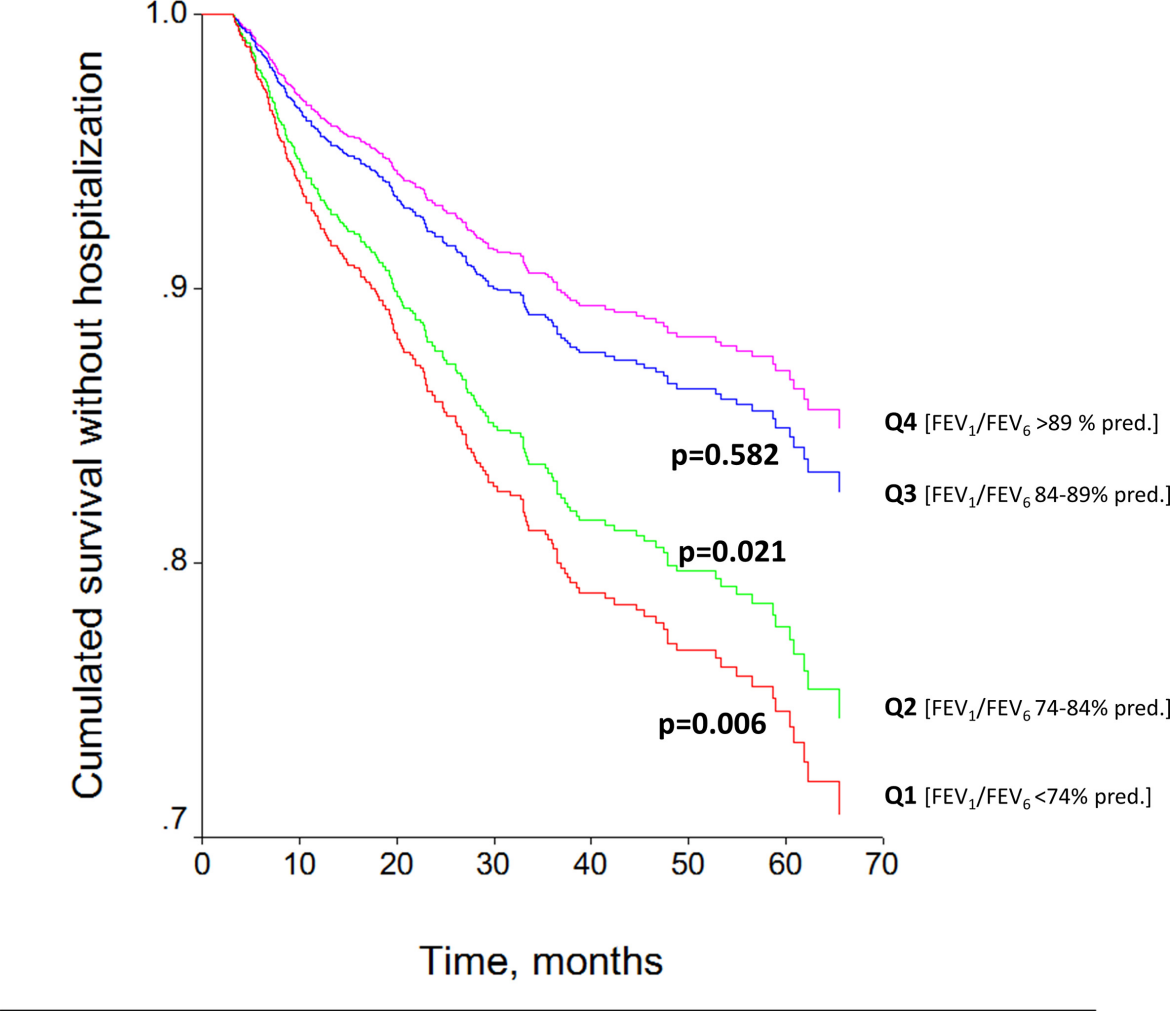
Adjusted risk for first hospitalization due to COPD exacerbation in patients classified according to quartiles of postbronchodilator FEV_1_/FEV_6_ ratio. Curves are adjusted for sex, age, BMI, smoking habit, Charlson morbidity index, current treatment and airflow limitation.

Eight hundred nine patients (30·9%) had at least one hospital admission due to COPD exacerbation during the follow-up period. The time to first admission was shorter for males, older patients, current or former smokers, and subjects with more morbidity, as well as in patients with more severe airflow limitation (Table 3). Lower values of postbronchodilator FEV_6_%pred. and FEV_1_/FEV_6_%pred. were also associated with a shorter time to first COPD admission during the follow-up period (Table 3). Interestingly, when all these variables were included in the stepwise Cox multiple regression model, sex, age, Charlson morbidity index, degree of airflow limitation, and quartiles of postbronchodilator FEV_1_/FEV_6_%pred. were retained as independent risk factors (Table 3, Fig 3).

**Table 3:** Risk factors for a first hospitalization due to COPD exacerbation.

		*Crude hazard ratio (95% CI)*	*p*	*Multivariate stepwise Cox regression*	
				*Adjusted hazard ratio* * *(95%CI)*	*p*
Males (vs. females)		3.232 (2.363–4.420)	<0.001	2.588 (1.299–3.577)	<0.001
Age, yrs.		1.037 (1.028–1.047)	<0.001	1.020 (1.002–1.037)	0.025
BMI, Kg/m^2^		0.995 (0.977–1.014)	0.609		
Smoking status			<0.001		
	Never smoker (n = 474)	1	-		
	Current smoker (n = 851)	2.598 (1.717–3.933)	<0.001		
	Former smoker (n = 1064)	3.949 (2.659–5.867)	<0.001		
Pack-years		1.013 (1.009–1.017)	<0.001		
Charlson morbidity index		1.192 (1.145–1.241)	<0.001	1.114 (1.020–1.218)	0.017
Airflow limitation severity (GOLD)			<0.001		<0.001
	Mild (n = 487)	1	-	1	-
	Moderate (n = 1267)	4.535 (2.223–9.251)	<0.001	2.921 (1.175–7.258)	0.021
	Severe (n = 451)	13.346 (6.561–27.144)	<0.001	5.566 (2.169–14.286)	<0.001
	Very severe (n = 75)	17.930 (8.440–38.089)	<0.001	7.288 (2.601–20.424)	<0.001
Postbronchodilator FEV_6_, % pred.		0.969 (0.963–0.975)	<0.001		
Postbronchodilator FEV_1_/FEV_6_, % pred.			<0.001		0.015
	Q4 (>0.89% pred.) (n = 571)	1	-	1	-
	Q3 (82–89% pred.) (n = 570)	1.069 (0.712–1.606)	0.747	1.172 (0.666–2.061)	0.582
	Q2 (74–84% pred.) (n = 570)	1.831 (1.274–2.632)	0.001	1.814 (1.096–3.002)	0.021
	Q1 (< 74% pred.) (n = 570)	3.864 (2.778–5.374)	<0.001	2.107 (1.232–3.603)	0.006

809 patients (30·9%) had at least one hospitalization due to COPD exacerbation during the follow-up period

*Adjusted for current treatment, BMI, smoking status, packs-year, postbronchodilator FEV_6_ (% pred.) and all variables included in the equation.

**Fig 4:**
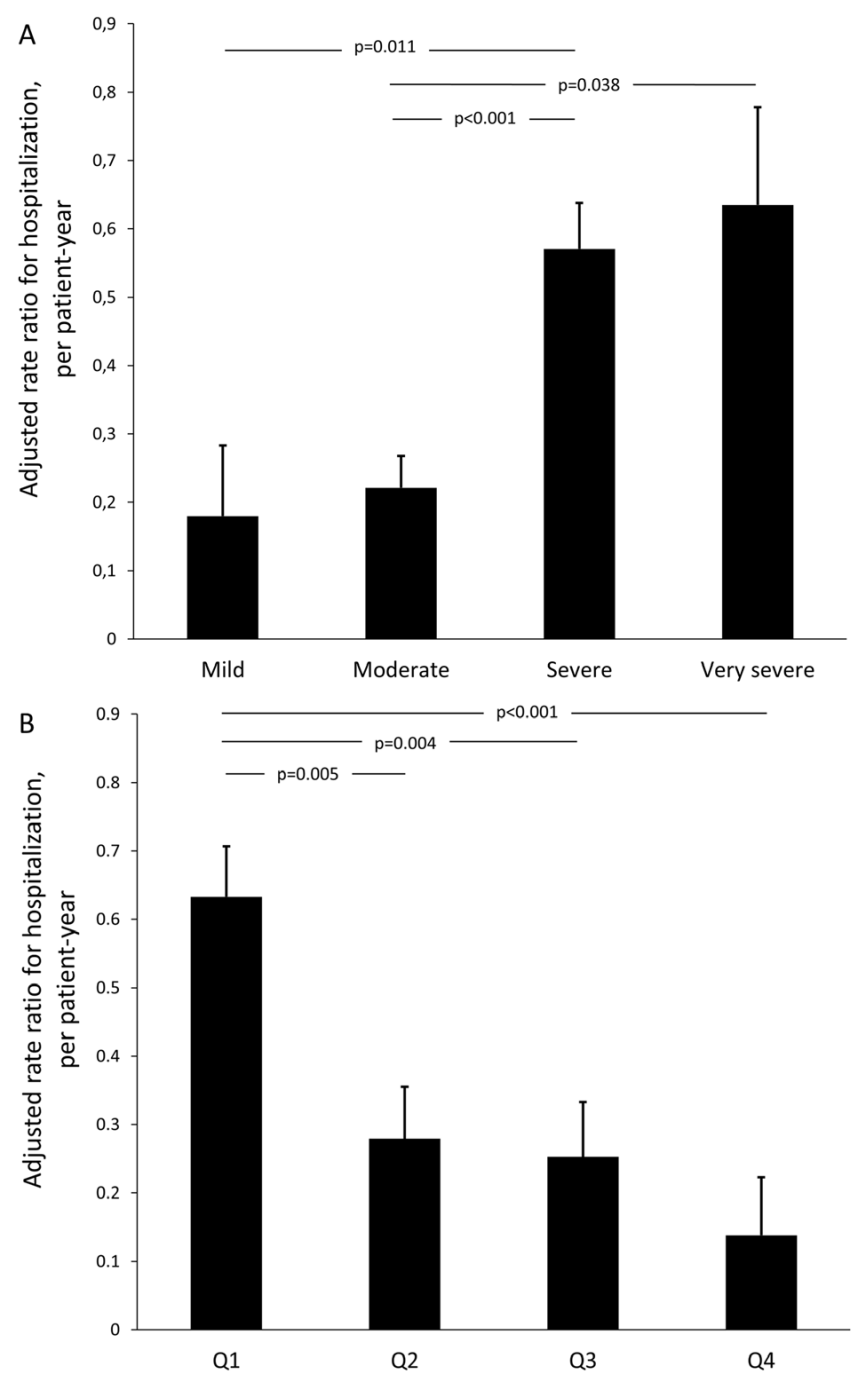
Comparison of adjusted weighted rate ratio for hospitalization by degree of airflow limitation (A) and quartiles of postbronchodilator FEV_1_/FEV_6_ ratio (B). Black boxes correspond to mean adjusted for sex, age, BMI, smoking status and Charlson morbidity index. Vertical lines represent standard error of the mean.

The weighted rate ratio for hospitalization was 0·28 (95CI: 0·23–0·33) per patient-year. This rate was higher in men than in women (0·36 ± 1·60 vs. 0·08 ± 0·25, *p*<0·05) and also in current or former smokers than in never smokers (0·22 ± 0·78 vs. 0·45 ± 1·95 vs. 0·08 ± 0·24, respectively; *p*<0·001). Moreover, the rate ratio for hospitalization due to COPD exacerbation was related to male sex, BMI, pack-years, Charlson morbidity index, and spirometric variables (S2 Table). After adjusting for confounding factors (sex, age, BMI, pack-years, and morbidity), differences in hospitalization rates were found between degrees of airflow limitation and quartiles of postbronchodilator FEV_1_/FEV_6_%pred. (Fig 4).

During the follow-up period, 220 COPD patients (8·4%) required hospitalization secondary to pneumonia, reaching a hospitalization rate ratio due to pneumonia of 0·05 per patient–year. Male sex, intensity of smoking history, comorbidity and current treatment with inhaled corticosteroids were identified as independent risk factors for hospital admission due to pneumonia. Postbronchodilator FEV_6_%pred. and FEV_1_/FEV_6_%pred., but not airflow limitation severity assessed by postbronchodilator FEV_1_%pred., were also identified as risk factors of pneumonia in our patients (S3 Table).

### Other outcomes

Lung function decline was evaluated in 1,713 patients with a mean interval between spirometries of 3 ± 1 year (range: 1–6). The mean loss of postbronchodilator FEV_1_ was 48 ml/year. A direct relationship was found between annual FEV_1_ decline and all spirometric parameters, including postbronchodilator FEV_6_%pred. (r = 0·312, p = 0·0002) and FEV_1_/FEV_6_%pred. (r = 0·102, p = 0·009). In the linear regression analysis, the only spirometric variable retained in the model to predict FEV_1_ decline was postbronchodilator FEV_6_%pred. (r^2^ = 0·069, p = 0·0002).

During the follow-up period, a new diagnosis of lung cancer was made in 145 patients (5·5%). In the multivariable logistic regression model, male sex, lower BMI, current smoking, Charlson morbidity index, and postbronchodilator FEV_6_%pred., and FEV_1_/FEV_6_%pred. were identified as independent risk factors (Table 4).

**Table 4:** Risk factors for a new diagnosis of lung cancer during the follow-up period*.

		*Multivariate odds ratio (95%CI)*	*P*
Males (vs. females)		3.748 (1.581–8.885)	0.003
BMI, Kg/m^2^		0.944 (0.992–1.043)	0.008
Smoking habit			0.131
	Never smoker (n = 474)	1	-
	Current smoker (n = 851)	2.905 (1.016–8.309)	0.047
	Former smoker (n = 1064)	2.757 (0.995–7.640)	0.051
Charlson morbidity index		1.237 (1.105–1.3859	0.0002
Airflow limitation severity (GOLD)			0.281
	Mild (n = 552)	1	-
	Moderate (n = 1448)	0.608 (0.233–1.588)	0.310
	Severe (n = 523)	0.360 (0.093–1.401)	0.141
	Very severe (n = 90)	0.196 (0.034–1.139)	0.069
Postbronchodilator FEV_6_, % pred.		0.976 (0.956–0.996)	0.018
Postbronchodilator FEV_1_/FEV_6_, % pred.			0.004
	Q4 (>0.89% pred.) (n = 571)	1	-
	Q3 (82–89% pred.) (n = 570)	1.334 (0.687–2.593)	0.395
	Q2 (74–84% pred.) (n = 570)	1.354 (0.693–2.648)	0.375
	Q1 (< 74% pred.) (n = 570)	3.276 (1.587–6.762)	0.001

During the follow-up period, a new diagnosis of lung cancer was made in 145 patients (5·5%).

*Multivariate logistic regression model adjusted for age and current treatment.

## Discussion

The main finding of the present study was that postbronchodilator FEV_6_ and FEV_1_/FEV_6_ as percentage of predicted were independent prognostic factors in stable outpatients with COPD. Postbronchodilator FEV_1_/FEV_6_%pred. and FEV_6_%pred., as well as male sex, older age and comorbidity, were the only variables independently associated with survival. Meanwhile, staging airflow limitation according to the original GOLD criteria, body mass index and pack-years did not yield any additional prognostic information. In addition to male sex, older age, comorbidity and FEV_1_%pred., FEV_1_/FEV_6_%pred. was also identified as an independent risk factor for hospitalization due to COPD exacerbation. Finally, FEV_6_%pred. was related to lung function decline, while FEV_1_/FEV_6_%pred. and FEV_6_%pred. were associated with a new diagnosis of lung cancer during the follow-up period.

The most outstanding contribution of our paper is to identify postbronchodilator FEV_1_/FEV_6_%pred. as an independent predictor of survival in a large, unselected general population of COPD outpatients. Several parameters were not independently associated with survival in the current study. Although FEV_1_ was higher among survivors, this difference was significant only in the univariate analysis. Several previous studies have also had findings similar to ours since they failed to find an association between FEV_1_ and survival in COPD [19,20].

Without a doubt, the first spirometric parameter that demonstrated prognostic capability was vital capacity, and several circumstances justify its validity in COPD patients. In addition to the severity of airflow obstruction represented by FEV_1_, in these patients it is important to consider the consequences of air trapping and lung parenchymal destruction, for which FVC could be an indirect indicator. In fact, in COPD patients, FVC reduction has been described in association with small airway collapse and air trapping [21]. Furthermore, the importance of comorbidities in COPD patients and their prognostic implications have been increasingly recognized over the last decade [22,23]. Several of these comorbidities, including diabetes, metabolic syndrome, heart failure, coronary disease, osteoporosis, hypertension, atrial fibrillation and muscular or hormonal disorders, affect spirometric values and are particularly related with reduced FVC [24,25]. As a surrogate parameter of FVC, it seems expectable that FEV_6_ could maintain a certain prognostic capability that could be even higher than FVC in patients with airflow limitation or elderly subjects, because FEV_6_ measurements are more easily achieved and more reproducible than FVC [6,10]. Although we do not have previous information in COPD patients, in elderly subjects treated at geriatric clinics for respiratory and nonrespiratory conditions, it has been reported that the mortality rate ratio was associated with having a low FEV_6_ [26].

At the same time, a decline in FVC secondary to COPD comorbidities, such as obesity, osteoporosis or heart failure, can induce a reduction in FEV_1_ disproportionate to the degree of airflow limitation, which would be partially compensated by the FEV_1_/FVC ratio. In fact, this ratio has shown advantages over FEV_1_ as an independent predictor for cardiovascular morbidity in patients with COPD, particularly of new episodes of ictus [27] or atrial fibrillation [25]. Also in this case, the FEV_1_/FEV_6_ ratio offers the advantage over the FEV_1_/FVC ratio of providing a more consistent and reproducible measurement, particularly if there is air trapping. Subjects with significant air trapping might reach and exceed their equal pressure point earlier and more peripherally before complete emptying and hence have FVC lower than expected for their age, creating a falsely high FEV_1_/FVC, a phenomenon that should be less likely to occur if FEV_6_ is used. Thus, Morris et al [28] describe that the FEV_1_/FEV_6_ ratio better identifies early anomalies in lung volumes or in diffusing capacity than FEV1/FVC, which is especially important since hyperinflation [29] as well as reduced diffusing capacity [30] are independent predictors of mortality in COPD.

Our data also show that the FEV_1_/FEV_6_%pred. is an independent predictor, in addition to FEV_1_%pred., for hospitalization due to COPD exacerbation, and is even more important in the case of hospitalizations due to pneumonia. This finding could partially agree with previous information that shows that the clinical deterioration of COPD is accompanied by a decline in the FEV_1_/FVC ratio at a greater magnitude than the fall in FEV_1_ [31]. Furthermore, in the ECLIPSE study, both FEV_1_ as well as the FEV_1_/FVC ratio were related with the development of exacerbations in the first year of follow-up [32], although the latter lost significance in the multivariate analysis. At the same time, other authors have reported that FEV_1_/FVC is an independent risk factor for the development of COPD exacerbation due to pneumonia [33].

As far as we know, there is no specific information about the value of the FEV_1_/FEV_6_ ratio for predicting COPD exacerbations. Nevertheless, it has a better correlation than FEV_1_/FVC with parameters that can contribute to exacerbation risk, such as dyspnoea, quality of life and exercise tolerance [34]. Moreover, FEV_1_/FEV_6_ predicts COPD-related structural disease on CT better than FEV_1_/FVC [34]. On volumetric CT scans of COPD patients, it has been observed to better correlate with the extension of structural damage (both air trapping as well as emphysematous areas), which can contribute to increase exacerbation risk while decreasing functional reserve given a respiratory infection.

Our study has several strengths and limitations. Among the former are the large number of patients included and the long follow-up period, including nearly 12,000 person–yrs. Second, the entire cohort was recruited in the same geographical area, and all clinicians followed the same COPD clinical guidelines for pharmacological and non-pharmacological treatment. Third, the follow-up information is very accurate, with few participants lost to follow-up. Several limitations, however, need to be acknowledged. Firstly, the patients were initially diagnosed with COPD on clinical grounds using a postbronchodilator FEV_1_/FVC ratio of <0·7. However, the lower limit of normal for this ratio was also employed as a selection criterion because it is considered a more reliable threshold for diagnosing airflow obstruction. Second of all, we did not discriminate between respiratory and non-respiratory mortality, nor did we consider other recognized risk factors, such as dyspnoea intensity or exacerbations, since our study was only focused on evaluating the prognostic value of different airflow limitations, which obviously should be considered together with other variables to construct multidimensional scales. Thirdly, in spite of the supposedly low diagnostic sensitivity of FEV_1_/FEV_6_ in patients with mild COPD [9], this variable maintains its prognostic capacity in patients with clinical confirmation of the COPD diagnosis. Lastly, all our participants were Caucasian with a clear predominance of males, reflecting the epidemiology of COPD in Spain [35]. Therefore, our results should be extrapolated to other populations with caution.

In conclusion, in a large, general COPD outpatient population, FEV_1_/FEV_6_%pred. (in quartiles) is an independent risk factor for both mortality as well as hospitalizations due to exacerbation. The demonstration of its prognostic value, in addition to its recognized capability to identify airflow limitation, provide this parameter with potential usefulness in both COPD diagnosis and severity classification.

## Supporting Information

S1 Table Characteristics of COPD patients according to survival. (DOC)

S2 Table Univariate linear regression models of predictors of rate ratio for hospitalizations due to COPD exacerbation. (DOC)

S3 Table Risk factors for hospitalization due to pneumonia during the follow-up period. (DOC)
