# Supplementary material for: Prognostic Value of the Six-Second Spirometry in Patients with Chronic Obstructive Pulmonary Disease: A Cohort Study
Source: PLoS One. 2015 Oct 21;10(10):e0140855. doi: 10.1371/journal.pone.0140855 (PMC4619273; doi:10.1371/journal.pone.0140855)
Supplement: S1 Table — (DOC) [file pone.0140855.s001.doc]

**Table S1**. Characteristics of COPD patients according to survival*

|  | | ***Survivors***  *(n=2517)* | ***Non survivors***  *(n=97)* | ***p*** |
| --- | --- | --- | --- | --- |
| Sex | | | | <0·001 |
|  | Males, % | 69·9 | 96·9 |
|  | Females, % | 30·1 | 3·1 |
| Age, yrs. | | 63 ± 12 | 72 ± 10 | <0·001 |
| BMI, Kg/m^2^ | | 28·9 ± 5·7 | 26·8 ± 4·5 | 0·001 |
| Smoking status | | | | <0·001 |
|  | Current smoker, % | 35·9 | 27·7 |
|  | Former smoker, % | 43·5 | 69·1 |
|  | Never smoker, % | 20·5 | 3·2 |
| Pack-years | | 47·3 ± 26·9 | 60·2 ± 29·4 | <0·001 |
| Charlson morbidity index | | 3·9 ± 2·1 | 5·3 ± 2·2 | <0·001 |
| Postbronchodilator FEV_1_, % pred. | | 60 ± 18 | 44 ± 16 | <0·001 |
| Postbronchodilator FVC, % pred. | | 73 ± 18 | 62 ± 17 | <0·001 |
| Postbronchodilator FEV_1_/FVC, % pred. | | 84 ± 13 | 73 ± 16 | <0·001 |
| Postbronchodilator FEV_6_, % pred. | | 79 ± 18 | 70 ± 19 | <0·001 |
| Postbronchodilator FEV_1_/FEV_6_, % pred. | | 81 ± 11 | 73 ± 13 | <0·001 |
| Positive reversibility test, % | | 30·9 | 32·0 | 0·468 |
| Airflow limitation severity (GOLD) | | | | <0·001 |
|  | Mild, % | 21·5 | 10·3 |
|  | Moderate, % | 56·1 | 38·1 |
|  | Severe, % | 19·0 | 45·4 |
|  | Very severe, % | 3·3 | 6·2 |
| Postbronchodilator FEV_6_ distribution | | | | <0·001 |
|  | Quartile 1 | 24·2 | 44·3 |
|  | Quartile 2 | 25·0 | 25·0 |
|  | Quartile 3 | 25·4 | 17·0 |
|  | Quartile 4 | 25·4 | 13·6 |
| Postbronchodilator FEV_1_/FEV_6_ distribution | | | | <0·001 |
|  | Quartile 1 | 23·8 | 53·4 |
|  | Quartile 2 | 25·0 | 25·0 |
|  | Quartile 3 | 25·6 | 9·1 |
|  | Quartile 4 | 25·5 | 12·5 |

* Data are presented as No. (%) or mean ± SD. Abbreviations: BMI=body mass index; FEV_1_=forced expiratory volume in 1 second; FVC=forced vital capacity; FEV_6_=forced expiratory volume in 6 seconds; VC=vital capacity.

P values were tested by Student t test if the variable is stated as mean ± SD or by chi-square test if the variable is stated as No. (%).
