# Supplementary material for: Prognostic Value of the Six-Second Spirometry in Patients with Chronic Obstructive Pulmonary Disease: A Cohort Study
Source: PLoS One. 2015 Oct 21;10(10):e0140855. doi: 10.1371/journal.pone.0140855 (PMC4619273; doi:10.1371/journal.pone.0140855)
Supplement: S2 Table — (DOC) [file pone.0140855.s002.doc]

**Table S2**. Univariate linear regression models of predictors of rate ratio for hospitalizations due to COPD exacerbation*

| **Parameter** | **R** | **95% Confidence Interval** | **P Value** |
| --- | --- | --- | --- |
| Age, yr | 0·104 | 0·066 – 0·142 | <0.001 |
| BMI, Kg/m^2^ | -0·051 | -0·089 – -0·013 | <0.001 |
| Pack-years | 0·098 | 0·053 – 0·143 | <0.001 |
| Charlson morbidity index | 0·113 | 0·075 – 0·151 | <0.001 |
| Postbronchodilator FVC, % pred. | -0·081 | -0·119 – -0·043 | <0.001 |
| Postbronchodilator FEV_1_, % pred. | -0·119 | -0·157 – -0·081 | <0.001 |
| Postbronchodilator FEV_1_/FVC, % pred. | 0·119 | 0·081 – 0·157 | <0.001 |
| Postbronchodilator FEV_6_, % pred. | -0·098 | -0·138 – -0·057 | <0.001 |
| Postbronchodilator FEV_1_/FEV_6_, % pred. | -0·123 | -0·163 – -0·082 | <0.001 |

*Data recorded are Pearson’s correlation coefficient, 95% confidence interval of the correlation and p value.

Abbreviations: BMI=body mass index; FVC=forced vital capacity; FEV_1_=forced expiratory volume in 1 second; FEV_6_=forced expiratory volume in 6 seconds.
