# Supplementary material for: Prognostic Value of the Six-Second Spirometry in Patients with Chronic Obstructive Pulmonary Disease: A Cohort Study
Source: PLoS One. 2015 Oct 21;10(10):e0140855. doi: 10.1371/journal.pone.0140855 (PMC4619273; doi:10.1371/journal.pone.0140855)
Supplement: S3 Table — (DOC) [file pone.0140855.s003.doc]

**Table S3**. Risk factors for hospitalization due to pneumonia during the follow-up period

|  | | ***Multivariate odds ratio*^*^ *(95%CI)*** | ***p*** |
| --- | --- | --- | --- |
| Males (vs. females) | | 2·731 (1·279–5·833) | 0·009 |
| BMI, Kg/m^2^ | | 0·979 (0·944–1·0145) | 0·250 |
| Pack-years | | 1·006 (1·001–1·012) | 0·031 |
| Charlson morbidity index | | 1·203 (1·083–1·336) | 0·001 |
| Airflow limitation severity (GOLD) | |  | 0·760 |
|  | Mild (n=552) | 1 | - |
|  | Moderate (n=1448) | 1·012 (0·347–2·948) | 0·982 |
|  | Severe (n=523) | 0·748 (0·193–2·894) | 0·674 |
|  | Very severe (n=90) | 0·763 (0·146–4·002) | 0·749 |
| Postbronchodilator FEV_6_, % pred. | | 0·979 (0·961–0·996) | 0·017 |
| Postbronchodilator FEV_1_/FEV_6_, % pred. | | - | 0·012 |
|  | Q4 (>89% pred.) (n=571) | 1 | - |
|  | Q3 (82–89 % pred.) (n=570) | 1·150 (0·610–2·170) | 0·666 |
|  | Q2 (74–84% pred.) (n=570) | 1·543 (0·852–2·793) | 0·152 |
|  | Q1 (< 74% pred.) (n=570) | 2·690 (1·395–5·186) | 0·003 |

During the follow-up period, 220 COPD patients (8·4%) required hospitalization secondary to pneumonia

*Multivariate logistic regression model adjusted for age and current treatment.
